# Description of a new species of *Rhinocoeta* Burmeister, 1842 (Scarabaeidae, Cetoniinae) from the South African Northern Cape

**DOI:** 10.3897/zookeys.848.34207

**Published:** 2019-05-20

**Authors:** Renzo Perissinotto

**Affiliations:** 1 School of Environmental Sciences, Nelson Mandela University, P.O. Box 77000, Port Elizabeth 6031, South Africa Nelson Mandela University Port Elizabeth South Africa

**Keywords:** Afrotropical region, Cetoniinae, new species, Northern Cape, *Rhinocoeta*, Xiphoscelidina

## Abstract

*Rhinocoetanamaqua***sp. nov.** is recognised as a separate species from its closest relative, *R.cornuta* (Fabricius, 1781) after a review and close analysis of specimens recently collected in the semiarid region of the Northern Cape Province, South Africa. The new species can be readily separated from *R.cornuta* by the drastically reduced tubercle and associated depression on its anterior pronotal margin, particularly in the male. In addition, the general body shape of *R.namaqua* is more globose than that of *R.cornuta*, its average total length is larger, and its elytral costae are generally reduced and poorly visible, particularly at the level of the umbones. These characteristics make it practically impossible to separate the two sexes of *R.namaqua*, without inspection of the internal reproductive organs, as their external morphologies are virtually identical, unlike in *R.cornuta*. Finally, the aedeagal parameres of *R.namaqua* exhibit a narrower apex than those of *R.cornuta* and, in particular, lack the subapical hook-shaped lateral expansions that are so typical of all the other *Rhinocoeta* s. str. species. The new species appears to be restricted to specific bioregions of the Succulent and Nama Karoo biomes of the Northern Cape, and like all other species of the genus is generally found on or under mammal herbivore dung. Adult activity is limited to short periods immediately after rainfall events, during which individuals fly around and mate, but do not feed on either fruits or flowers.

## Introduction

The genus *Rhinocoeta* Burmeister, 1842 currently includes two subgenera, the nominal one with five recognised species and R. (Haematonotus) Kraatz, 1880 with three species (Holm and Marais 1992, Beinhundner 2017). On the basis of adult and larval morphology, it has been argued that the genus may phylogenetically be placed close to the subtribe Xiphoscelidina rather than the Cetoniina, and that it constitutes part of a relict lineage derived directly from the most primitive Cetoniinae (Krikken 1984, Holm 1992, Smith et al. 1998). However, preliminary genetic DNA analyses seem to indicate that it may actually be closer to the more modern Cetoniina than previously believed (Kouklík 2017, Šípek et al. 2016). Consequently, their perceived “relictual/primitive” characters may actually represent adaptations to their peculiar habitat (e.g., droughts, extreme seasonality, scarce vegetation) and to their unique lifestyle, characterized by short bursts of activity and inability to feed at the adult stage (Smith et al. 1998).

Close analysis of a series of specimens collected during the past two decades in the Namaqualand and Upper Karoo regions of the Northern Cape, South Africa, has revealed that a new species previously confused with *R.cornuta* (Fabricius, 1781) occurs in these semiarid regions. None of the four synonyms used in the past to refer to *R.cornuta* can in fact be associated with the populations of the western part of the Northern Cape that constitute the new species. High resolution photos, collection data and circumstantial evidence obtained from the ZMUK (Kiel, Germany), BMNH (London, UK), MNHN (Paris, France), have led to the conclusion that *Cetoniacornuta* Fabricius, 1781, *Scarabaeusarcas* Olivier, 1789, *Cetoniacornigera* Gmelin (in L.), 1790 and *Scarabaeushispidolugubris* Voet, 1779 all fit the typical characteristics of the Cape south-western populations of *R.cornuta*. This is also consistent with the period of their description, the late 18^th^ century, which coincided with the early exploration of the subcontinent. It would have been virtually impossible for the collectors of that period to have ventured beyond the colonial settlement of Cape Town and its immediate surroundings on the south and west coasts. Indeed, the remote Northern Cape populations remain very poorly sampled even in the modern era, with only approximately 20 specimens currently known for the new species here described.

Extensive observations made recently throughout the southern African region have also allowed for a better resolution of the distribution range and, particularly, the ecology of all the species of the nominal subgenus Rhinocoeta. This has prompted a reanalysis of the taxonomic position of *R.limbaticollis* (Péringuey, 1907), a rather enigmatic “species” described at the turn of the 19^th^ century on the basis of one female specimen only.

## Materials and methods

Specimens for this study were obtained through direct collections in the field during the period 1996–2018 (R Perissinotto and L Clennell legit), or from museum and private collections (as per list provided below). Fresh specimens were either caught in flight using standard nets after rainfall events, or collected on or under dung accumulations of a variety of herbivorous mammal species. Holotype, lectotype, paratypes, and other specimens of *R.cornuta* were analysed from high-resolution photographic material submitted by the museum curators listed in the Acknowledgement section.

For the description of morphological characters, the terminology used by Krikken (1984) and Holm and Marais (1992) is followed in this study. Specimen total length and maximum width were measured using a Vernier calliper, from the anterior margin of the clypeus to the apex of the pygidium and at the widest point of the elytra, respectively. Photos of specimen dorsal and ventral habitus were taken with a Nikon CoolPix S9700 digital camera with macro setting, while photos of the male genitalia were obtained using a Nikon DigitalSight DS-Fi2 camera attached to a Nikon SMZ25 dissecting microscope. The background was removed from the photos using Microsoft Word 2010 (Picture Tools), in order to increase clarity of resolution. The Combine ZP Image Stacking Software by Alan Hadley (alan@micropics.org.uk) was used to obtain z-stacking composite images.

### Repositories are abbreviated as follows:

**BMPC** Jonathan Ball and Andre Marais Private Collection, Cape Town, South Africa

**BMNH** Natural History Museum, London, United Kingdom

**BMSA** The National Museum, Bloemfontein, South Africa

**GBPC** Gerhard Beinhundner Private Collection, Euerbach, Germany

**ISAM** Iziko South African Museum, Cape Town, South Africa

**ISNB** lnstitut Royal des Sciences Naturelles, Brussels, Belgium

**MNHN** Muséum National d’Histoire Naturelle, Paris, France

**SANC** South African National Collection of Insects, Pretoria, South Africa

**TGPC** Thierry Garnier Private Collection, Montpellier, France

**TMSA**Ditsong National Museum of Natural History (formerly Transvaal Museum), Pretoria, South Africa

**ZMUK** Zoologisches Museum der Christian-Albrechts-Universität zu Kiel, Kiel, Germany

Data on distribution, period of adult activity, and other biological information for *R.cornuta* were also obtained from Péringuey (1907), Holm (1992), Allard (1991), Sakai and Nagai (1998) and Beinhundner (2017). The key abbreviations for the provinces of the Republic of South Africa used within the text are as follows:

**EC** Eastern Cape

**FS** Free State

**NC** Northern Cape

**WC** Western Cape

## Taxonomy

### Rhinocoeta namaqua sp. nov.

Taxon classificationAnimaliaColeopteraScarabaeidae

http://zoobank.org/AB13CDDD-DE3E-48F7-81C1-D1202F91E6A3

Figures 1 , 3 , 5

#### Diagnosis.

**Figure 1.**
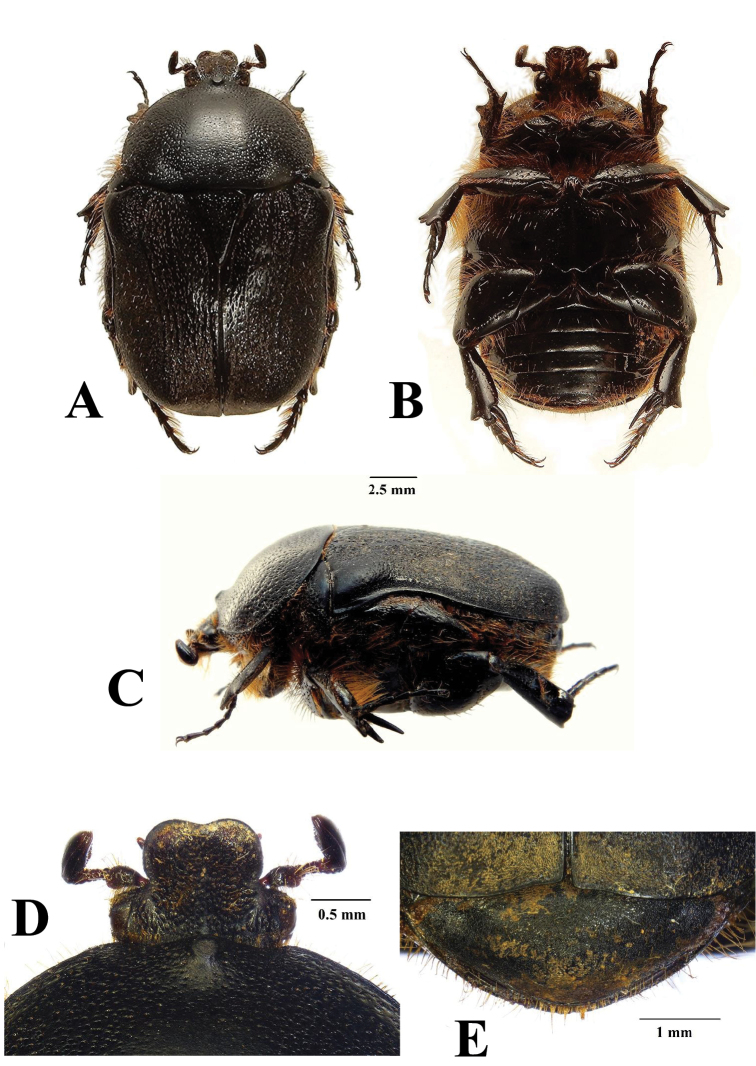
*Rhinocoetanamaqua* sp. nov: dorsal (**A**) ventral (**B**) and lateral (**C**) views of body habitus, with details of clypeus (**D**) and pygidium (**E**). Photographs by Lynette Clennell.

**Figure 2.**
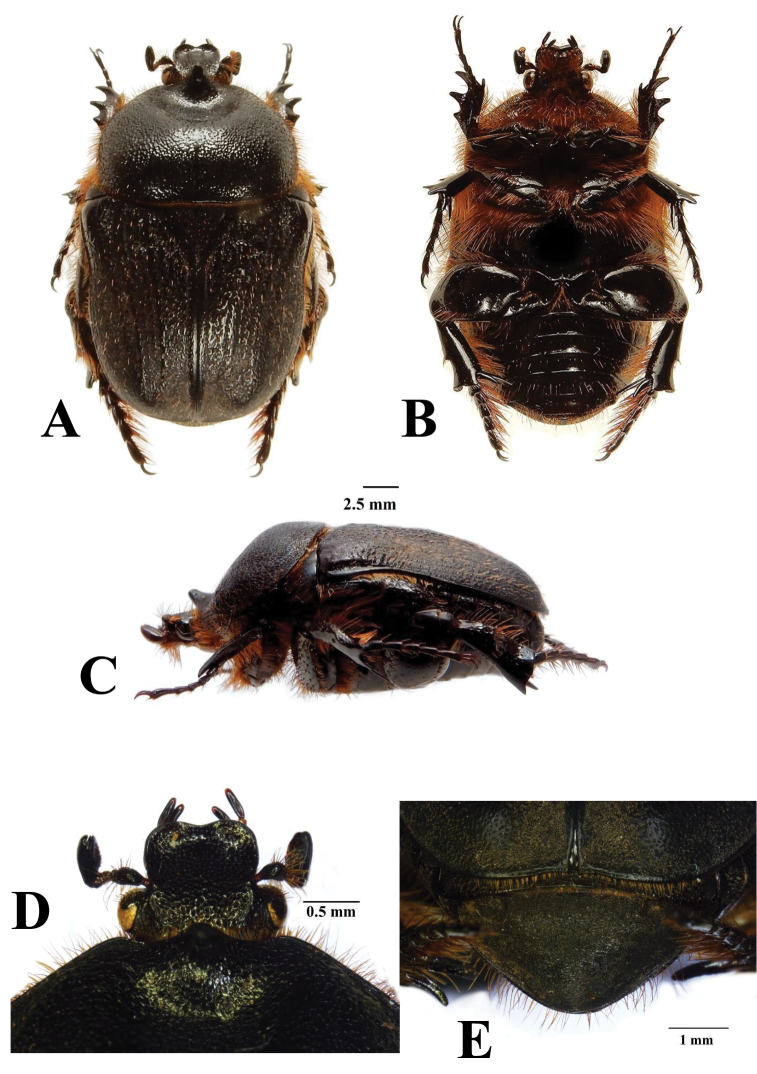
*Rhinocoetacornuta* (Fabricius, 1781): dorsal (**A**) ventral (**B**) and lateral (**C**) views of body habitus, with details of clypeus (**D**) and pygidium (**E**). Photographs by Lynette Clennell.

**Figure 3.**
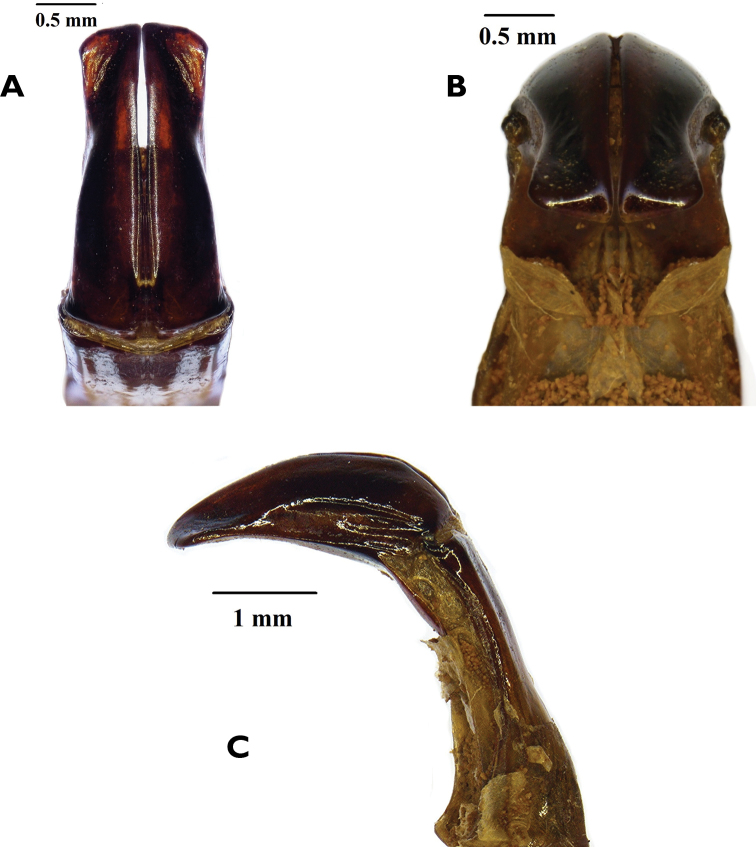
*Rhinocoetanamaqua* sp. nov: dorsal (**A**) frontal (**B**) and lateral (**C**) views of male aedeagus Photographs by Lynette Clennell.

**Figure 4.**
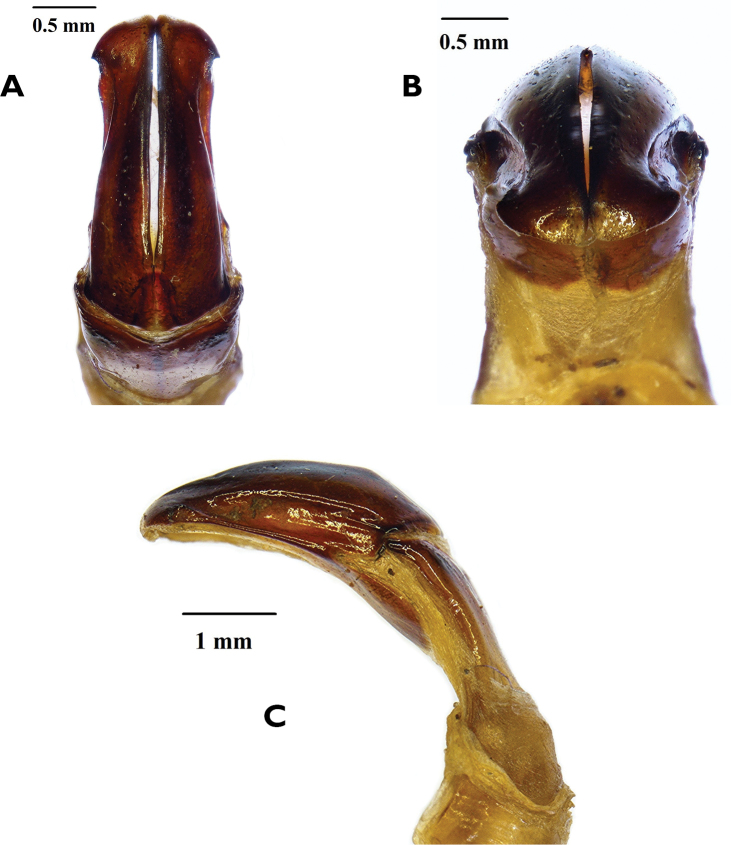
*Rhinocoetacornuta* (Fabricius, 1781): Dorsal (**A**) frontal (**B**) and lateral (**C**) views of male aedeagus. Photographs by Lynette Clennell.

The two species can be separated mainly on the basis of the male parameres, which in *R.namaqua* lack the spine-like expansions on the apico-lateral margins that are so typical of the parameres of *R.cornuta* (compare Figs 3 and 4).The new species can also be recognised by the virtual absence of the depression behind the pronotal tubercle, which on the other hand is very prominent in *R.cornuta*, especially in the male (compare Figs 1 and 2). The pronotal tubercle of *R.namaqua* is very short and blunt in both sexes, with the apical surface virtually flat (Fig. 1A). Conversely, in *C.cornuta* this is generally quite elevated in the male and in both sexes the apex is generally smoothly rounded (Fig. 2A). Essentially in *R.namaqua* it is very difficult, if not impossible, to separate males from females on the basis of external morphology alone, as the pronotal tubercle is similarly reduced and the associated depression is lacking in both sexes. These characters are, on the other hand, key towards the separation of the sexes in *R.cornuta*.

Further to this, the two closely related species also exhibit differences at the level of the elytral costae three and five which, with some notable exceptions, are largely obsolete in most specimens of *R.namaqua* but still noticeable in *R.cornuta*, at least in the proximal two thirds of the elytra, above the apical umbone (compare Figs 1 and 2). Additionally, the body shape of *R.namaqua* is remarkably more globose and its average length larger than that of *R.cornuta*, i.e., 20.3–24.6 mm versus 12.5–22.5 mm (Holm 1992, Beinhundner 2017), respectively.

#### Description of holotype male

(Figs 1A–E, 3A–C). *Size*. Length 22.2; width 14.1 mm.

*Body*. Completely black and generally matte, with small shiny areas restricted to elytral suture, basal portion of costae, humeral callus and peri-scutellar area (Fig. 1A); globose with dense sculpture throughout dorsal surface and short, scattered yellowish setae on dorsal periphery, becoming longer and denser on lateral margins (Figs 1A, C, D).

*Head*. Black with dense but coarse sculpture throughout surface and poorly elevated vertical, median ridge on frons; with medium to long yellow-brown setae on frons, eye canthus and antennal pedicel and basal margin; clypeus bilobate and deeply concave, with lateral margins elevated but straight posteriorly and smoothly rounded anteriorly; antennal clubs black to dark brown, of normal cetoniine length, slightly longer than flagellum; pedicel black but flagellum dark brown.

*Pronotum*. Completely black, matte and virtually semicircular in shape, with apically flat tubercle at anterior margin and smooth angles at postero-lateral margins, forming straight line in front of scutellum; peritubercular depression poorly developed and barely noticeable; small, scattered round punctures on disc, becoming larger, denser and more elongate towards lateral and anterior margins; short, scattered yellow-brown setae on lateral and anterior declivities, becoming longer and denser at all margins except posterior (Figs 1A, C).

*Scutellum.* Black, isoscelic triangular with sharply pointed apex and deep but narrow lateral grooves; with scattered round to elongate punctures across the surface and short setae on basal margin only (Fig. 1A).

*Elytron*. With costae barely visible and shiny around sutural, periscutellar area and two basal thirds of third and fifth costae; rest of surface matte and densely sculptured with round to vertically elongate punctures, becoming rugose on lateral and apical declivities; with short, erect tawny-coloured setae scattered regularly across whole surface, except periscutellar area and umbones; with apices smoothly rounded and matching perfectly at sutural joint, without significant gap or spinal projections; both humeral and apical calluses pronounced (Figs 1A, B).

*Pygidium*. Uniformly black, broadly elliptical with dense and fine rugose sculpture; with moderate central convexity and shallow, symmetric baso-lateral depressions; bearing thin, long setae along entire apical margin, with denser cluster around apical point.

*Legs*. Short and robust, with typical fossorial adaptations; tarsal segments moderately developed but tibiae thickened and expanded laterally, with several spurs, spines and denticles; protibia tridentate, with third tooth substantially reduced; mesotibia short, reinforced with mid outer ridge, three apical spines and two spurs; metatibia short but extremely robust, with supporting diagonal outer ridge, one hypertrophic spade-like spine and two thick spurs, with proximal spur hypertrophic and reaching half distance of total metatarsal length (Figs 1A, B, C); femora equally robust and expanded, reaching hypertrophy in metalegs.

*Ventral surface*. Black and shiny, but overwhelmingly covered in long, dense tawny-coloured setae, except on ventral side of femora, metasternum and abdominal sternites; exhibiting small and sparse round sculpture throughout surface; mesometasternal lobe smoothly rounded and poorly protruding anteriorly, with regularly spaced round punctures and thin setae emerging at their centre; abdominal sternites flat to very slightly depressed around middle.

*Aedeagus*. Parameres with dorsal lobes laterally expanded, covering completely ventral lobes in dorsal view (Fig. 3A); exhibiting constriction towards apical third, then expanding again at apex; apex flattening abruptly, with lateral corners sharp, but not exhibiting spinal protrusion (Figs 3A, B); duck-bill shaped and smoothly curved in lateral view (Fig. 3C).

#### Description of female.

Superficially, there is virtually no sexual dimorphism in this species, as its male lacks the deep depression around the pronotal tubercle, and the tubercle itself is normally short and blunt. This contrasts markedly with *R.cornuta*, where the male typically exhibits a well-developed tubercle (often hypertrophic) surrounded by a wide and deep depression on the anterior margin of the pronotum (Figs 2A, C, D). As a result, males and females of *R.namaqua* can only be separated by using a suite of secondary characters, especially the generally protruding pygidium and the slightly more convex abdominal sternites in the latter sex. The meso- and metatibial spurs are also substantially shorter in the female than in the male counterpart, particularly the proximal ones. The female is also more deeply and densely sculptured on the dorsal area, particularly on the pronotum, where small round punctures are uniformly distributed across its surface.

#### Distribution.

**Figure 5.**
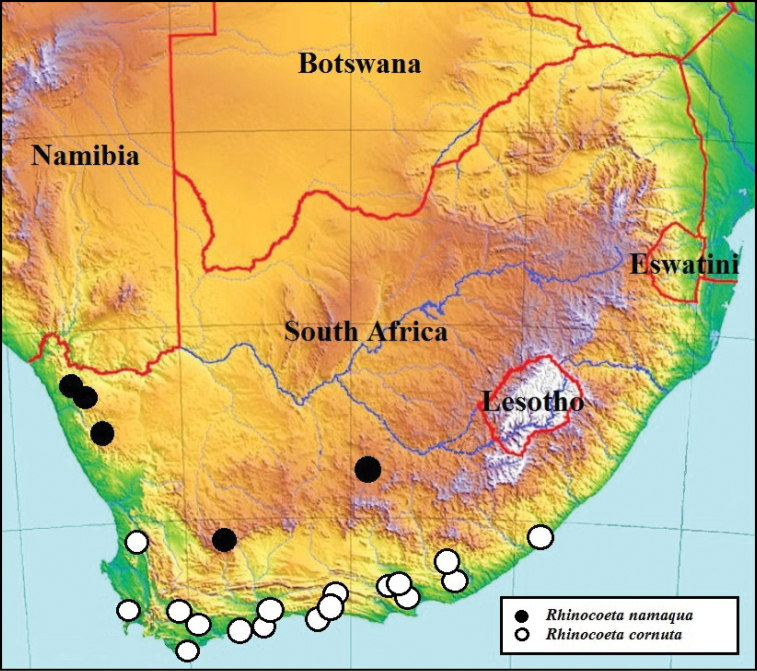
Known distribution range of *Rhinocoetacornuta* (Fabricius, 1781) and *Rhinocoetanamaqua* sp. nov. within southern Africa (Map adapted from Mapsland: Copyright 2019 Mapsland).

All known records are from areas situated above the South African Great Escarpment, in the Succulent and Nama Karoo biomes of the Northern Cape Province (Fig. 5). The specific bioregions included in its range are the Namaqualand Hardeveld, the Trans-Escarpment Succulent Karoo and the Upper Karoo, respectively (Mucina and Rutherford 2006). Thus, the species appears to be a specialist of arid to semiarid environments.

#### Biology.

Larval development seems to be linked to sandy soils, generally in or around dry riverbeds or in alluvional or erosion deposits. Adults have often been collected in or under dung hills of herbivore mammals, including farmed goats and sheep. Its life cycle, therefore, appears to be similar to that of other, better-known members of the genus *Rhinocoeta*, e.g., *R.sanguinipes* (Smith et al. 1998), although its larval stages remain undescribed. Adult activity seems to be restricted to the austral summer, from December to March, and emergence from the soil has been observed to be linked to rainfall events (pers. obs.). No adult specimen has yet been recorded feeding, either on flowers, fruits or tree sapping flows and, thus, it is almost certain that their period of adult activity may be very short and sustained only by energy reserves accumulated during larval development (Smith et al. 1998, Perissinotto et al. 1999).

#### Derivatio nominis.

The species is named after the semiarid Namaqualand region of South Africa (Northern Cape Province), where most known specimens were collected.

#### Remarks.

There is no variability in the colouration within the series of specimens examined in this study. However, the elevation of elytral costae three and five varies substantially among specimens, with most exhibiting poorly elevated to obsolete costae, but a minority showing pronounced costae (e.g., paratype from De Aar). Within the type series, the size ranges as follows: ♂ length 20.3 – 24.2 mm, width 13.2 – 14.4 mm (n = 10); ♀ length 23.3 – 24.6 mm, width 13.4 –14.6 mm (n = 6).

#### Type material.

Holotype (♂): South Africa, NC, Goegap Nat Res, 30 Dec 1996, R Perissinotto & L Clennell (ISAM). Paratypes: 5♂♂ + 4♀, as above (BMPC); 1♂, South Africa, Northern Cape, Sutherland, Swaarweerberg 1683 m, 32°23'50.1"S, 20°34'39.3"E, 01 Jan 2008, AP Marais leg (BMPC); 3♂♂ + 2♀, South Africa, NC, Kamieskroon, 26 Jan 2018, R Perissinotto & L Clennell; 1 ind, South Africa, Northern Cape, Anenous, Namaqualand, 01/01/1911, CL Biden leg (ISAM: COL-A027336); 1♀, S.W. Africa, De Aar (50 milles au N d’Upington), 7-III-1950, H-J Brédo (ISNB: R.I.Sc.N.B., I.G. 18.284) [Reference to SW Africa and distance from Upington most likely erroneous; A Drumont, pers. comm.].

### Updated and expanded identification key to the species of *Rhinocoeta* s. str. (revised after Holm 1992)

**Table:** 

1	Body black, often with some brick-red areas; elytra sculptured with well-discernible round to crescent-shaped punctures	**2**
–	Body never bicolorous; elytra between costae finely and densely textured, without discernible crescent sculpture	**3**
2	Scutellum with punctures elongate; pronotal punctures round; underside, pronotal sides and legs brick-red, but in westernmost populations entirely black; length: 17.5 – 24.9 mm; distribution: South Africa (WC, EC, NC, FS) and southwestern Namibia	**R. (R.) sanguinipes Gory & Percheron, 1833**
–	Scutellum with round punctures; pronotal punctures crescent-shaped; body completely black or pronotal sides and elytral disc with variable degree of brick-red colouring; length: 19.5 – 24.0 mm; distribution: South Africa (EC, NC – central Karoo mountains, at altitudes > 1500 m)	**R. (R.) maraisi Holm, 1992**
3	Pronotum with tubercle at middle of anterior margin, not forming depression around it; third and fifth elytral costae converging at apical umbone; length: 10.0 – 16.0 mm; distribution: South Africa (WC, NC, EC, FS), unconfirmed old records also in Namibia and Zimbabwe	***R.armata* Boheman, 1860**
–	Pronotum with prominent to moderate tubercle at middle of anterior margin, forming shallow depression to deep concavity around it; third and fifth elytral costae weakly elevated and becoming obsolete before reaching apical umbone	**4**
4	Antero-median pronotal tubercle and associated depression showing marked sexual dimorphism, becoming hypertrophic and deep in male; aedeagal paramereres with latero-apical spinal expansion; length: 12.5 – 22.5 mm; distribution: South Africa (WC, EC – coastal lowlands and Cape Fold mountains) (Fig. 5)	**R. (R.) cornuta (Fabricius, 1781)**
–	Antero-median pronotal tubercle and associated depression poorly developed and similar in both sexes; aedeagal paramereres without latero-apical spinal expansion; length: 20.3 – 24.6 mm; distribution: South Africa (NC – Namaqualand, Roggeveld and Upper Karoo) (Fig. 5)	**R. (R.) namaqua sp. nov.**

## Discussion

The new description reported here brings to six the total number of species currently recognised within the nominal subgenus: *R.sanguinipes* (Gory & Percheron, 1833); *R maraisi* Holm, 1992; *R.armata* Boheman, 1860; *R.limbaticollis* (Péringuey, 1907), *R cornuta* (Fabricius, 1781) and *R.namaqua* sp. nov. (Holm 1992, Beinhundner 2017). There are then three further species in the subgenus Haematonotus Kraatz, 1880: R. (H.) turbida (Boheman, 1860), R. (H.) hauseri (Kraatz, 1896) and R. (H.) leonardi Beinhundner, 2013 (Beinhundner 2017). These have adequately been dealt with and thoroughly illustrated recently by Beinhundner (2013, 2017).

**Figure 6.**
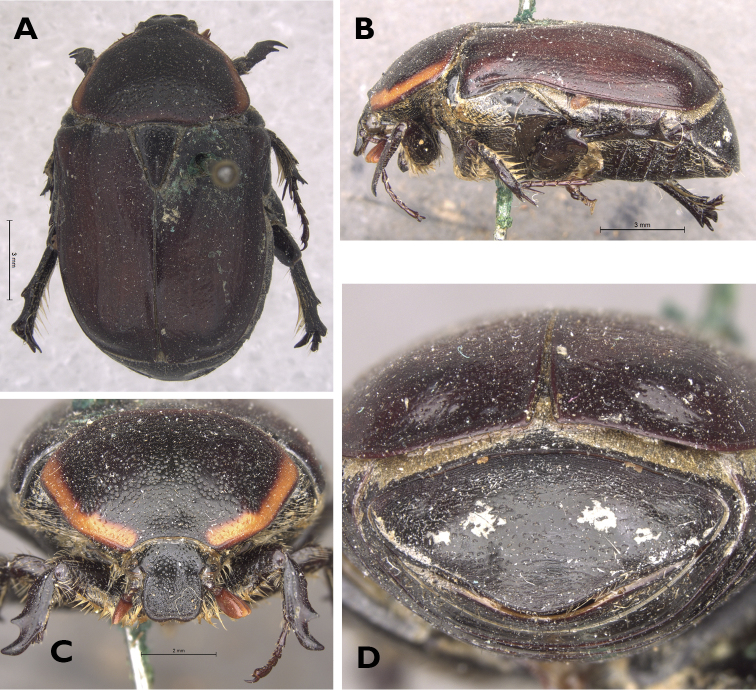
*Rhinocoetalimbaticollis* (Péringuey, 1907), Holotype ♀ **A** dorsal habitus **B** lateral view **C** head and pronotum **D** pygidium. Photographs by Aisha Mayekiso; copyright Iziko Museums of South Africa.

The issue of *R.limbaticollis*, however, remains a complex and unresolved one. It was described under a different genus, *Lipoclita*, by Péringuey (1907) mainly on the basis of its uncharacteristic mouthparts. These were regarded as fundamental by Péringuey (1907), but have subsequently been downgraded by Holm (1992), on the basis that mouthparts are variable and regressive, particularly in taxa such as all the members of the genus *Rhinocoeta* s. l. that do not feed at the adult stage. A close re-analysis of its key features reveals that *R.limbaticollis* largely resembles a typical dark/black female of R. (H.) turbida particularly in terms of protibiae, clypeus, antennal clubs and mesosternal process (Figs 6A–D), yet differs from this in terms of two key characters. These are: 1) the presence of cretaceous spots on the pygidium (Fig. 6D; although these are very regressive and not as developed as in males R. (H.) turbida); and 2) the lack of dense and coarse (even rugose on the pronotum) sculpture on the dorsal aspect (Fig. 6A).

Considering that this is unfortunately still the only specimen known for this “species”, despite the extensive searches that were undertaken in the area of the type locality during the past 30 years, there seem to be only two options left regarding its identity. The first is that it indeed represents a separate species to R. (H.) turbida, very rare or even extinct, as suggested by Holm and Marais (1992). This is difficult to believe, because the broader area of its type locality has not been completely “transformed” by mining and agriculture. There are in fact still protected and virtually pristine reserves in that area (e.g., Vaalbos/Mokala National Park, Sandveld Nature Reserve, Vredefort Dome World Heritage Site). Also direct observations made on virtually all the species of this genus indicate that they are very adaptable and resilient to anthropogenic activities (pers. obs.). The second option is that the specimen represents a case of gynandromorphy of R. (H.) turbida, with a mixture of female (e.g., bidentate protibiae, convex abdomen) and male characters (small and scattered sculpture, cretaceous spots on pygidium). It is hoped that the formulation of these working hypotheses may trigger further research, both in the field and the lab, to finally resolve this issues with conclusive evidence. To facilitate this, high-resolution photos of the specimen are presented here for the first time, courtesy of ISAM Collections Manager, Aisha Mayekiso.

Since the revision of the genus *Rhinocoeta* by Holm (1992) and Holm and Marais (1992), much has been learned about the biology/ecology and geographic distribution of the five confirmed species now constituting the nominal subgenus. Smith et al. (1998) reported that adults of the genus are generally collected under dung pads of cattle or in dung middens of a large variety of indigenous antelopes. In the Winterberg range of the Eastern Cape, larvae, pupae, and eclosing adults of *R.sanguinipes* have been found under a bontebok (*Damaliscusdorcasdorcas*) dung midden (Smith et al. 1998). Still in the Eastern Cape, but in the Sneeuberg, adults of *R.maraisi* sdults were collected in cattle dung (Holm and Stobbia 1995).

More comprehensive investigations carried out in the last 20 years have revealed that the four larger species, i.e., *R.sanguinipes*, *R.maraisi*, *R.cornuta* and *R.namaqua*, all depend on herbivorous and insectivorous mammal dung for their development (pers. obs.). On the other hand, the larva of *R.armata* has been observed making subsurface tunnels in sandy soil, in order to drag detrital matter underground, including leaf litter and pieces of dung pellets, of kudu (*Tragelaphusstrepsiceros*) for instance (pers. obs., P Malec and P Šípek, pers. comm.). *Rhinocoetasanguinipes* has been observed most frequently in large dung accumulations of klipspringer (*Oreotragusoreotragus*), but also in smaller dung deposits of aardvark (*Orycteropusafer*), red hartebeest (*Alcelaphusbuselaphuscaama*) and even domesticated goats, sheep and cows. *Rhinocoetamaraisi* seems to prefer dung droppings of mountain reedbuck (*Reduncafulvorufula*), Cape hare (*Lepuscapensis*) and farmed horses and cows. *Rhinocoetacornuta* on the Western and Eastern Cape south coast thrives on dung of farmed goats and sheep, but also of bontebok, hartebeest and other unidentified antelopes. Finally, the new species, *R.namaqua*, has so far been observed only on dung droppings of kudu and farmed sheep (pers. obs., J Ball and AP Marais, pers. comm.).

In terms of distribution, further to the ranges already reported in Holm (1992), Holm and Marais (1992) and Holm and Stobbia (1995), new data show that, apart from the Sneeuberge, *R.maraisi* occurs also in other major mountains of the Eastern and Northern Cape Karoo, such as the Bamboesberg, the Kikvorsberg and the Groot Tafelberg. The more widespread distribution of *R.sanguinipes* is confirmed, with populations occurring across the south-western and central parts of South Africa, as well as in southern Namibia. As previously reported by Holm (1992), the Namibian population exhibits entirely black ventral and dorsal habitus, and it has now been established that the westernmost South African populations also follow this colour pattern, generally lacking the typical brick-red pigmentation on ventral surface, legs, pygidium and pronotal lateral margins. Concerning *R.armata*, however, no evidence has been obtained in confirmation of the dubious distribution records of this species in Zimbabwe and Namibia, thus suggesting that the old references to these localities may be incorrect, as proposed earlier by Holm and Marais (1992). Finally, *R.cornuta* and *R.namaqua* seem to be clearly separated in their distribution by the Great South African Escarpment, with the first species restricted to the coastal lowlands and Cape Fold Mountains below it, while the second species has so far only been recorded in the north-western highlands above the escarpment (Fig. 5).

## Supplementary Material

XML Treatment for Rhinocoeta namaqua
